# Unravelling the genome of the brackish water malaria vector *Anopheles aquasalis*

**DOI:** 10.1038/s41598-023-47830-1

**Published:** 2023-11-22

**Authors:** Rodrigo Maciel Alencar, Cesar Camilo Prado Sepulveda, Luis Martinez-Villegas, Ana Cristina Bahia, Rosa Amélia Santana, Igor Belém de Souza, Gigliola Mayara Ayres D’Elia, Ana Paula Marques Duarte, Marcus Vinicius Guimarães de Lacerda, Wuelton Marcelo Monteiro, Nágila Francinete Costa Secundino, Paulo Filemon Paolucci Pimenta, Leonardo Barbosa Koerich

**Affiliations:** 1 Fundação de Medicina Tropical Dr. Heitor Vieira Dourado, Manaus, Amazonas CEP 69.040-000 Brazil; 2 Programa de Pós-Graduação em Medicina Tropical, Fundação de Medicina Tropical Heitor Vieira Dourado, Universidade do Estado do Amazonas, Manaus, Amazonas CEP 69.040-000 Brazil; 3 Instituto de Pesquisas René Rachou, Fundação Oswaldo Cruz, Belo Horizonte, Minas Gerais CEP 30.190-009 Brazil; 4 Programa de Pós-Graduação em Ciências da Saúde, FIOCRUZ, Belo Horizonte, Minas Gerais CEP 30.190-009 Brazil; 5 Laboratório de Bioquímica de Insetos e Parasitos, Instituto de Biofísica Carlos Chagas Filho, Universidade Federal do Rio de Janeiro, Rio de Janeiro, CEP 21.941-170 Brazil; 6 Instituto de Pesquisa Leônidas & Maria Deane, Fundação Oswaldo Cruz, Manaus, Amazonas CEP 69.027-070 Brazil; 7 University of Texas Medical Branch, Galveston, United States of America; 8 Departamento de Parasitologia, Universidade Federal de Minas Gerais, Belo Horizonte, Minas Gerais CEP 31.270-901 Brazil

**Keywords:** Genome evolution, Entomology

## Abstract

Malaria is a severe public health problem in several developing tropical and subtropical countries*. Anopheles aquasalis* is the primary coastal malaria vector in Central and South America and the Caribbean Islands, and it has the peculiar feature of living in water with large changes in salinity. Recent research has recognised *An. aquasalis* as an important model for studying the interactions of murine and human *Plasmodium* parasites. This study presents the complete genome of *An. aquasalis* and offers insights into its evolution and physiology. The genome is similar in size and gene content to other Neotropical anophelines, with 162 Mb and 12,446 protein-coding genes. There are 1387 single-copy orthologs at the Diptera level (eg. *An. gambiae*, *An. darlingi* and *Drosophila melanogaster*). *An. aquasalis* diverged from *An. darlingi*, the primary malaria vector in inland South America, nearly 20 million years ago. Proteins related to ion transport and metabolism belong to the most abundant gene families with 660 genes. We identified gene families relevant to osmosis control (e.g., aquaporins, vacuolar-ATPases, Na+/K+-ATPases, and carbonic anhydrases). Evolutionary analysis suggests that all osmotic regulation genes are under strong purifying selection. We also observed low copy number variation in insecticide resistance and immunity-related genes for all known classical pathways. The data provided by this study offers candidate genes for further studies of parasite-vector interactions and for studies on how anophelines of brackish water deal with the high fluctuation in water salinity. We also established data and insights supporting *An. aquasalis* as an emerging Neotropical malaria vector model for genetic and molecular studies.

## Introduction

Malaria is a severe public health problem in several tropical and subtropical areas, mostly in countries in Africa, Asia, and America. It is a leading cause of death and disease in many developing countries, where young children and pregnant women are most affected^1^. Mosquitoes in the genus *Anopheles* are vectors of human malaria parasites, *Plasmodium* sp., which annually generate approximately 229 million cases that result in nearly half a million deaths worldwide^1^. Parasites are transmitted through the bite of a female mosquito of the *Anopheles* genus. However, although this genus comprises 400 species, only 41 recognised as vectors, nine of which are found in the Americas^2^. The biological characteristics, influenced by variations in the ability of *Anopheles* vectors to transmit the parasite (e.g., molecular components of the immune response, microbiota, and intestinal physiology), are well studied and have been characterised in established models such as *Anopheles gambiae* and *Anopheles stephensi*^3–6^.

Publication and availability of *Anopheles* genomes accelerated research that has enhanced our fundamental understanding of mosquito genetics, behaviour, physiology, and roles in transmission and can contribute to new strategies for combating malaria^7^. Although our knowledge that genome information will facilitate the development of innovative approaches to combat malaria and other mosquito-borne diseases, until today, only two Neotropical malaria vectors have had their genomes sequenced and are publicly available: *Anopheles albimanus* and *Anopheles darlingi*.

*Anopheles aquasalis* is among the species considered important in malaria transmission in the New World and is the primary coastal vector in Central and South America and the Caribbean Islands^8,9^. It is considered the primary *Plasmodium vivax* malaria vector from northeastern Venezuela to southern Brazil^10,11^, where its larvae develop in the brackish waters of mangroves. *An. aquasalis* is among the few anophelines capable of surviving in severe water salinity changes. Rising sea levels due to climate change have raised concerns regarding a greater risk of disease transmission in coastal regions^12^. However, few studies have addressed how anopheline larvae deal with saline stress. Physiological studies have suggested that morphological changes in the localisation of vacuolar-ATPases (V-ATPases) and K+/Na+ ATPase proteins are essential for osmotic regulation in *An. albimanus*^13,14^. Other molecular and transcriptome studies have also suggested that other genes, such as aquaporin and carbonic anhydrases, in conjunction with transcriptional modulation, are essential for mosquito survival in hyperosmotic or hypo-osmotic environments^15–18^.

Besides its peculiar ecological features as the primary coastal malaria vector, recent research has recognised *An. aquasalis* as an essential model for studying the interaction with human *Plasmodium* and murine parasites such as *P. yoelii*^19–21^. As such, it has been possible to identify and functionally characterise the role of molecular components relevant during the mosquito invasion by *Plasmodium* species^19,22–24^. Our understanding of the interactions between *An. aquasalis* and the malaria parasites are rapidly improving. Thus *An. aquasalis* is emerging as a model species for genetic and molecular studies.

However, despite its vectorial, ecological, and modelling importance, few genomic studies on *An. aquasalis* have been carried out to date. Genomic studies are fast and reliable methods for the genome exploration of medically necessary non-model insects^25,26^. Genomic and transcriptomic studies have provided a better understanding of the genetic characteristics of more than 18 anopheline species. They have established the composition of conserved regions of genes, the identification of highly divergent genes, the recognition of gene families, and the evolution of species-specific physiological or behavioural genetic variations^24,27–29^. Here, we present the analysis of the genome of *An. aquasalis* as a resource and platform for fundamental and translational studies. By identifying its protein-coding genes, we uncovered insights into genome evolution, structure, proteins relevant to osmotic regulation, insecticide resistance, and genes pertinent to vector-parasite and vector-host interactions, among others.

## Results

### Genome assembly and annotation of *An. aquasalis*

**Figure 1:**
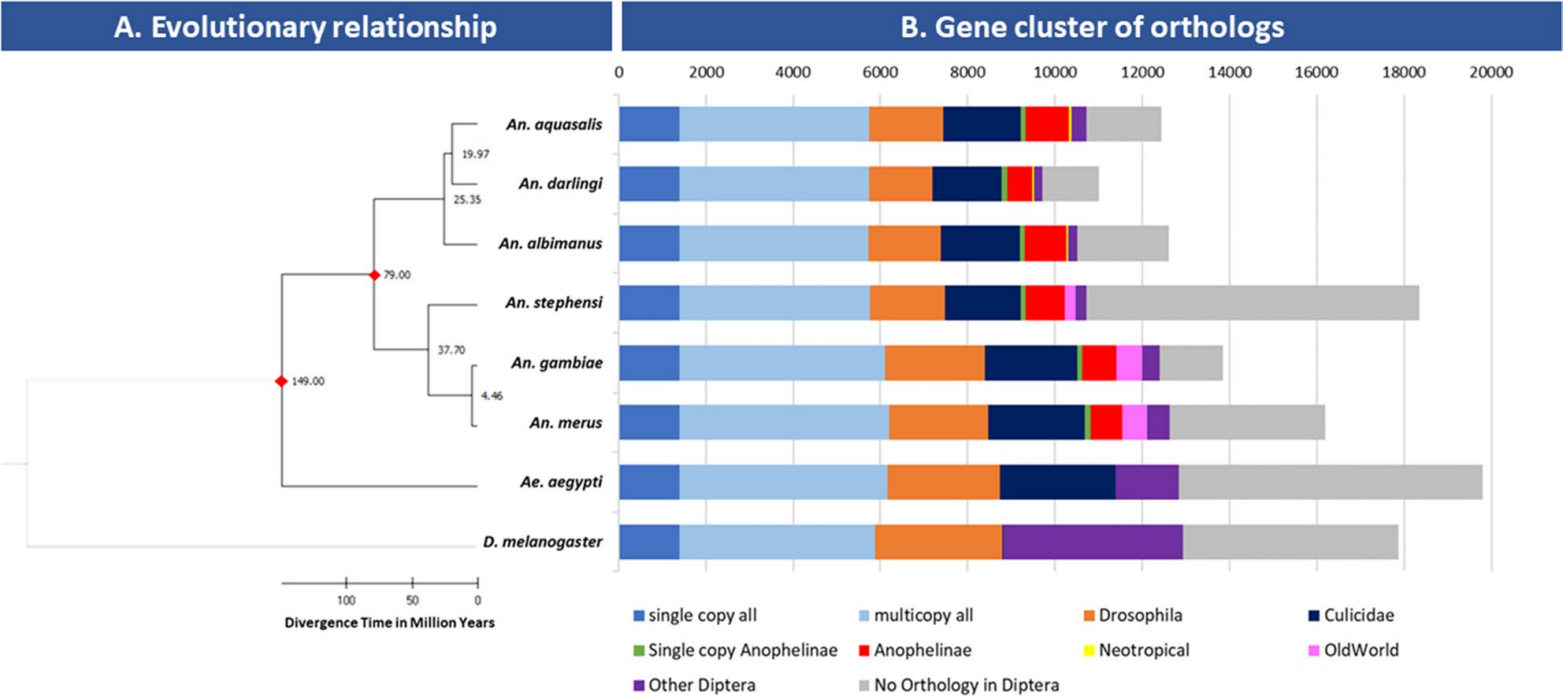
Evolutionary and orthology analysis of Neotropical anophelines. (**A**) The species phylogeny tree was inferred based on 1387 single-copy orthologs at Diptera level and rooted on *Drosophila melanogaster*. Divergence times were calculated using two fixed calibration constraints (red diamonds). (**B**) Bars show the orthology of annotated genes in each taxon. Single copy all: single copy orthologs present in all taxa; multicopy all: multicopy orthologs present in all taxa; *Drosophila*: orthologs present in *Drosophila melanogaster* and at least one mosquito; Culicidae: orthologs present in *Ae. aegypti* and at least one anopheline; Single-copy Anophelines: single-copy orthologs present in all anophelines only; Anophelinae: orthologs present in more than one anopheline (but not all); Neotropical: orthologs present only in Neotropical anophelines; Old World: orthologs present only in African and Asian anophelines; Other: genes with orthology with other Diptera; No Orthology: genes that presented no orthology at Diptera level.

The genome sequencing generated ~ 123 million 100b paired-end reads (SRA SRX21970089). The assembled *An. aquasalis* genome (Accession GCA_002846955.1) spans 162,944 Mb, distributed on 16,504 scaffolds (N50 14,431). A total of 12,446 protein-coding genes were annotated in the genome. Benchmarking Universal Single-Copy Orthologues (BUSCO) analysis for genome [94.6% of complete single-copy genes, 0.2% complete and duplicated, 1.9% fragmented and 3.3% missing genes] and for annotation [92.1% of complete single-copy genes, 0.3% complete and duplicated, 2.2% fragmented and 5.4% missing genes] shows similar quality when compared to other mosquitoes (Additional file 1: Figure S1). The gene structure models generated with the MAKER program were evaluated according to the annotation edit distance (AED), with most gene structures supported by evidence, with 90% having a value of between 0 and 0.5 for AED (Additional file 1: Figure S2). Orthology analysis comparing *An. aquasalis* with *An. darlingi, An. albimanus, An. gambiae, An. merus, An. stephensi, Aedes aegypti,* and *Drosophila melanogaster* (Fig. 1B) identified 1387 single-copy orthologs (SCO) among the compared genomes; 4359 genes that are multicopy; 1766 genes exclusive to mosquitoes; 121 genes that are single-copy only in anophelines; 992 genes are present in at least one other Anophelinae, 55 genes that are exclusive to Neotropical anophelines; 345 genes with orthology to other Diptera and 1721 genes that presented no orthology at Diptera level. Based on the 1387 SCO, we reconstructed the evolutionary tree of *An. aquasalis* and other Neotropical Anophelinae and calculated the divergence times for each branch (Fig. 1A). The phylogenetic tree suggests that *An. aquasalis* diverged from *An. darlingi* ~ 19.97 million years ago (mya), while South American anophelines diverged from *An. albimanus* ~ 25.35 mya.

Gene cluster analysis (Additional file 1: Figure S3) of orthologous genes suggests that 3154 genes are specific to *An. aquasalis* when compared to *An. darlingi, An. albimanus* and *An. gambiae.* A cluster of 7012 orthologs are present in all four anophelines, while 331 orthologs are present only in Neotropical anophelines. The mean transcript size was 4059,96 bp, while coding sequences presented a mean length of 420.88 bp (Additional file 1: Figure S4). In all, 35,352 introns were identified in the genome of *An. aquasalis* with an average size of 666.45 bp. Finally, the average gene size was predicted to be 3508.37 bp (Additional file 1: Table S1).

Repetitive element analysis suggests that ~ 6.3% of *An. aquasalis* genome is composed of such elements (Table 1). The most abundant classes were Interspaced Repeats (3.1%) and simple repeats (2.4%). Transposable elements accounted for 0.62% of the genome, and the most representative families were Jockey, Gypsy, Bel/Pao and Mariner/TC1 (Additional file: Figure S5).

**Table 1:** Repetitive Element identification in the *An. aquasalis* genome.

Repetitive element class	Subclass	Number of elements	Length (BP)	% seq
Transposable elements				
SINEs	Alu	33	6075	0.004%
	Others SINE	36	5908	0.004%
	Others LINE	5996	490,975	0.301%
LINES	LINE1	73	4118	0.003%
	LINE2	136	26,889	0.017%
	L3/CR1	117	17,933	0.011%
LTR	Others LTR	2385	457,214	0.281%
	ERV Class I	6	383	0.000%
	ERV Class II	24	1457	0.001%
Repetitive DNA elements		10,094	1,388,204	0.852%
Unclassified		25,547	2,742,549	1.683%
Interspased repeats			5,085,017	3.121%
RNA small		63	4446	0.003%
Simple repeats		121,702	3,976,717	2.441%
Low complexity repeats		9266	415,557	0.255%
Total		44,510	10,231,168	6.279%

### Functional prediction of encoded genes from the *An. aquasalis* genome

**Figure 2:**
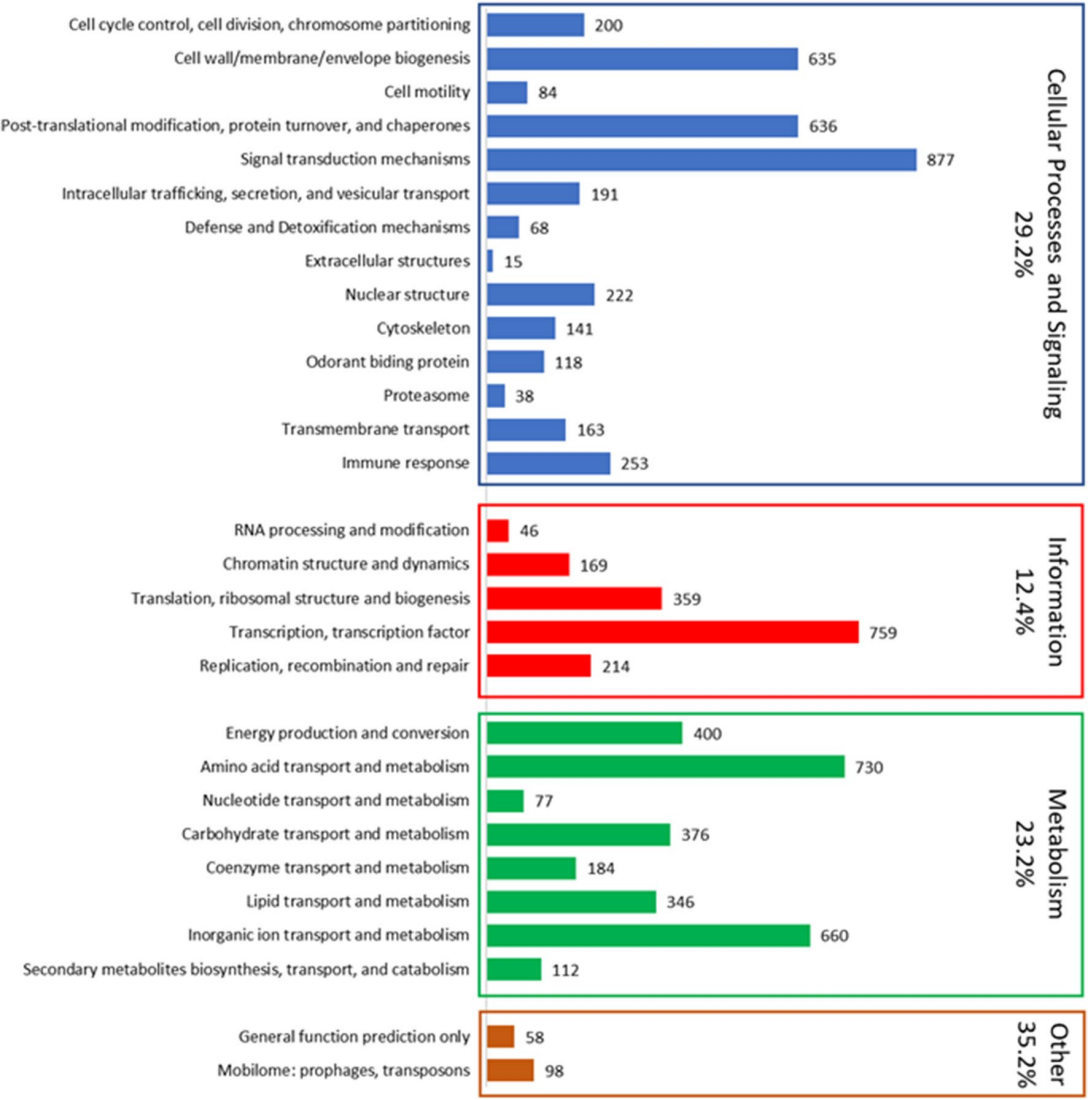
Summary of annotated genes in the *An. aquasalis* genome. Bars represent the number of genes annotated in each functional class. Colours represent major functional groups, and the percentage of genes in each category is represented. The percentage of genes belonging to the category of other genes (orange) includes genes with unknown functions (4217 genes not represented by a bar in the figure due to scalability).

The putative functions were inferred for 65.9% (8208) of the predicted protein-coding genes (Fig. 2; Additional file 2). From the genes with identified ontologies and putative functions, our analysis indicated that most terms corresponded to the category of cellular processes and signalling (29.2%), followed by metabolism with 23.2%, and information storage and processes (12.4%) (Fig. 2). Looking at the more specific classification, the most abundant classes of genes belonged to signal transduction mechanisms (877 genes); transcription and transcription factors (759 genes); amino acid transport and metabolism (730 genes); inorganic ion transport and metabolism (660 genes); post-translational modification, protein turnover, and chaperones (636 genes) and cell wall/membrane/envelope biogenesis (635 genes). Altogether, the genes in these six functional classes encompass 51.7% of all genes with ascertained putative function. As expected, most annotated genes have unknown functions (4242 genes: 34.1%), though 98 genes were classified into the mobilome functional class (transposons and prophages).

The analysis of the composition of domains with the InterproScan tool recognized among the main families of most representative proteins those that are composed of the domains zinc finger C2H2-type (IPR013087) with 301 proteins, the zinc finger, RING/FYVE/PHD-type (IPR013083) with 196 proteins and zinc finger, RING-type (IPR001841) with 101 proteins. Domains related to catalytic processes, such as protein kinase domains (IPR000719) with 212 proteins and serine proteases and trypsin domain (IPR001254) with 200 proteins, also had considerable representation. Other well-represented domains were those related to cell recognition processes, DNA repair, which is part of cell surface receptors and the immune response, such as immunoglobulin-like domain (IPR007110) with 168 proteins, leucine-rich repeat, typical subtype (IPR003591) with 122 proteins, leucine-rich repeat (IPR001611), RNA recognition motif domain (IPR000504) with 120 proteins and fibronectin type III (IPR003961) with 64 proteins. Finally, some groups of domains related to the structure of the cuticle were abundant, such as insect cuticle protein (IPR000618) with 97 proteins and the chitin-binding domain (IPR002557) with 96 proteins (Additional file 1: Table S3). In the next section, some groups of interest are discussed.

### Osmoregulation, ion metabolism and transport

**Figure 3:**
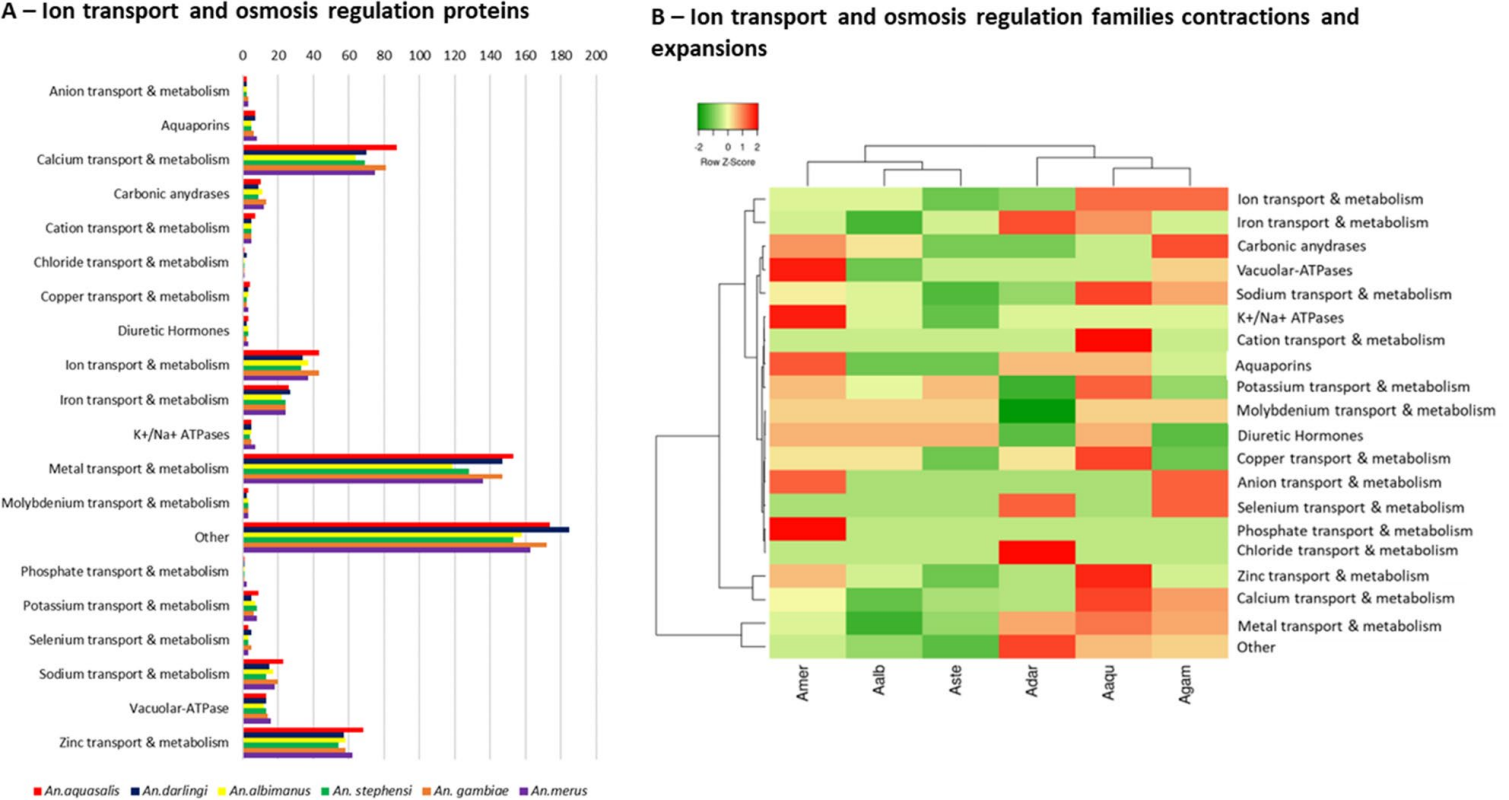
Ion metabolism and transport and osmosis proteins in *An. aquasalis*. (**A**) shows the number of genes related to ion metabolism and transport found in *An. aquasalis* in comparison to other anophelines. (**B**) Heatmap of expansions (red) and contractions (green) of gene families in anophelines.

*An. aquasalis* larvae live in brackish waters, and osmoregulation is a crucial process for these anophelines, involving ion transport and metabolism genes, water permeability, and tissue modifications. We found 685 proteins related to ion transport and metabolism in *An. aquasalis*, which is higher than other anophelines, particularly Neotropical anophelines (Fig. 3A; Additional File 3—Dataset 2). The *An. aquasalis* genome presented a higher number of Cation transport proteins, zinc, copper, sodium and calcium transport and metabolism proteins (Fig. 3A; Additional File 3—Dataset 2).

**Figure 4:**
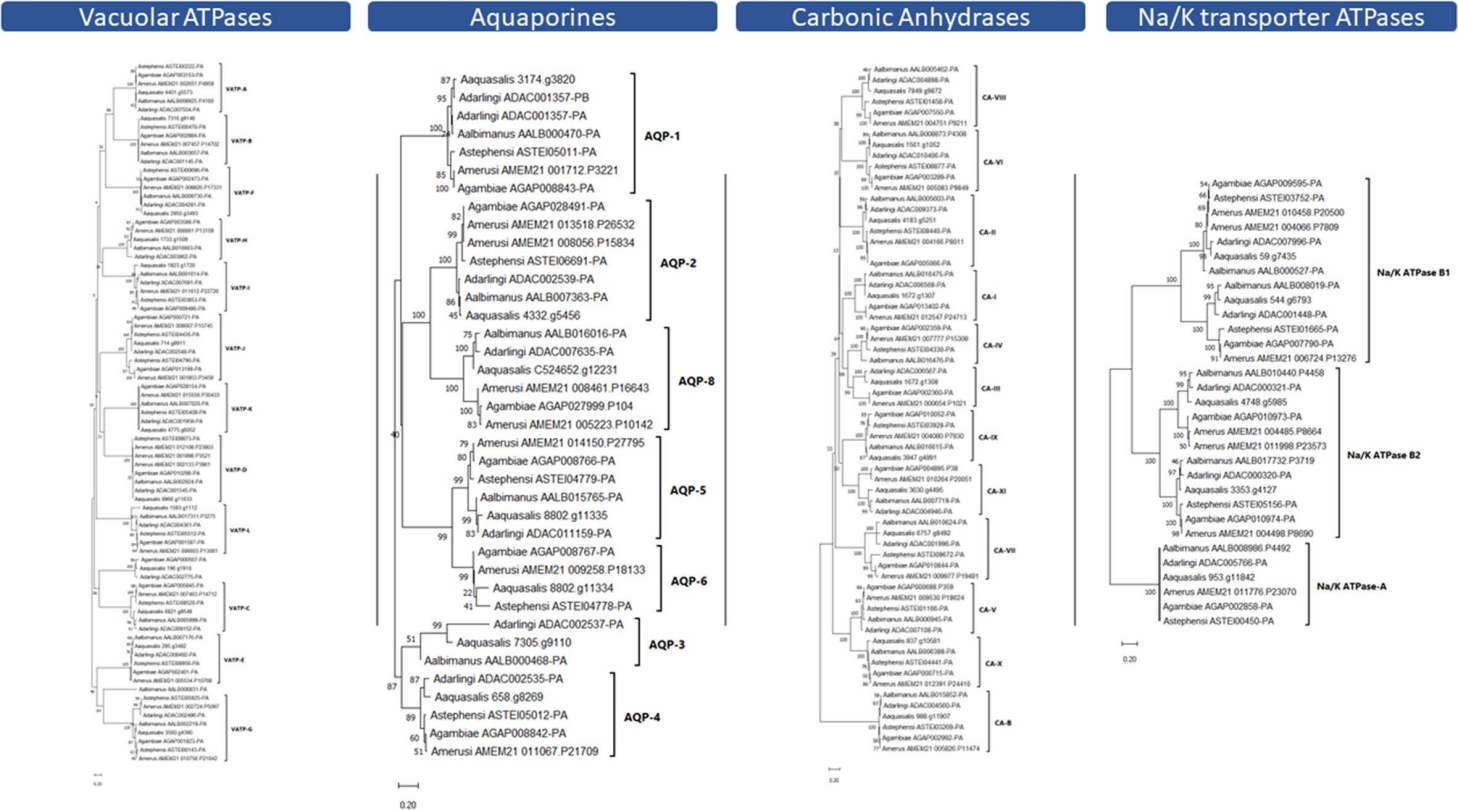
Evolutionary relationships of the osmotic regulation genes. The evolutionary relationships of *An. aquasalis* (Aaq); *An. darlingi* (Adar); *An. albimanus* (Aalb) *An. merus* (Amer) and *An. gambiae* (Agam) osmotic regulation genes. (**A**) The Vacuolar ATPases proteins; (**B**) Aquaporins; (**C**) Na/K ATPases and; (**D**) Carbonic Anhydrase. Trees were inferred by neighbour-joining, using amino acid sequences, and bootstrapped (10,000 replicates).

Studies of the tolerance of *An. albimanus* to saltwater also suggests the importance of V-type ATPase (V-ATPase), carbonic anhydrase, and K+/Na+ ATPase proteins in osmoregulation^13–17^, and studies of other insects have shown the role of aquaporins in this process. Orthology searches suggest that *An. aquasalis* has a similar, but slightly lower, number of osmoregulation genes (Figs. 3B and 4) compared to other anophelines, especially *An. merus*. Heatmap analysis on the number of genes in osmoregulation and ion transport/metabolism (Fig. 3B) shows that *An. aquasalis* has a significantly higher number of cation transporters (7 genes while the mean is 5.3 ± 0.8 for other anophelines) and zinc transport/metabolism (68 genes while the mean is 59.5 ± 4.9 for other anophelines), while *An. merus* has a significantly higher number of phosphate transport/metabolism (two copies instead of one for all other anophelines) and canonical osmoregulation genes such as vacuolar ATPsaes (16 genes while the mean is 13.5 ± 1.4) and K+/Na+ ATPases (7 genes while the mean is 5.1 ± 1.0).

Evolutionary analyses and codon-based tests (Fig. 4; Additional file 3—Dataset 3) suggest that almost all osmotic regulation genes evolve via strong purifying selection. The significantly higher number of *An. merus* V-ATPases seem to originate from multiple duplication events in the V-ATPase D family (Fig. 4), while the increase in the K+/Na+ ATPases of *An. merus* originates from two duplications in the B1 and B2 families. Purifying selection was observed even between gene subfamilies (e.g., all seven aquaporin gene subfamilies), except for vacuolar ATPases, in which evolutionary analysis suggests purifying selection within subfamilies but not between subfamilies (e.g., VATP-F and VATP-G).

### Immune response genes

Identifying genes involved in the mosquito responses against *Plasmodium* sp. Is particularly interesting for understanding parasite-vector interactions. Hence, genes of the immune response system in *An. aquasalis* were discussed in depth in a companion paper^30^, and here we will briefly present the most important findings and insights. We identified 278 immune-related proteins, divided into 24 families or signalling pathways groups, in the *An. aquasalis* genome. All genes from the classic signalling pathways; Janus kinase (JAK)/signal transducer and activator of transcription (STAT), immune deficiency (Imd), Toll and Jun N-terminal kinase (JNK)—were identified with one-to-one orthologs for *An. darlingi.* Cascade modulators (e.g., serine proteases) accounted for 25.64% of identified immune response genes. Signalling pathways genes correspond to 13.46%. Other abundant families in the immune response group are Fibrinogen-Related Proteins (FREPs) with 29 genes (9.29%), autophagy process with 20 genes (6.41%), leucine-rich repeats (20; 6.41%) and C-type lectins (13; 4.16%). In general, *An. aquasalis* has a similar number of immune response genes to other Neotropical anophelines (*An. darlingi* 294; *An. albimanus* 304); but a significantly lower number of genes when compared to *An. gambiae* (410).

### Chemosensory system

Anophelines use a series of chemosensory proteins to perceive the environment they are in, such as identifying hosts and oviposition sites. Chemosensory genes are classified as chemosensory proteins (CSPs) and odorant binding proteins (OBPs). They can be divided into three families of significant importance: odorant receptors (ORS), gustatory receptors (GRs), and ionotropic receptors (IRs). Our analysis found 44 ORS, 32 GRs, and 15 IRS in the *An. aquasalis* genome is a lower number than all anophelines studied so far, including *An. darlingi*, with 57 ORS and 56 GRs. Despite the low conservation of OBP amino acid sequences, we identified six conserved cysteine residues characteristic of this gene family. All *An. aquasalis* OBPs were also classified into Classic, Atypical, and Plus-C subfamilies according to their homology to *An. gambiae* sequences and phylogeny (Additional file 1: Figures S6, S7 and Table S4). Motif analysis identified eight conserved motifs in OBPs, with conserved cysteine residues in four (Additional file 1: Figure S8).

### Insecticide resistance and detoxification

In all, 133 genes were identified related to metabolic detoxification, 73 (54%) from the P450s family (Table 2), 25 glutathione-S-transferases (GST), and 36 carboxylesterases (Additional file 1: Table S5 and Table S6). Evolutionary analyses with *An. gambiae* P450 genes (Additional file 1: Figure S9, S10 and S11) allowed us to classify *An. aquasalis* P450 into four classic clans: CYP2 (8 genes), CYP3 (32 genes), CYP4 (25 genes), and mitochondrial CYP (8 genes). These numbers were similar to the genes found in the genome of *An. darlingi*; however, *An. albimanus* and *An. gambiae* presented a greater number of genes.

**Table 2:** *An. aquasalis* cytochrome P450 gene subfamilies compared with *An. darlingi*, *An. albimanus* and *An. gambiae*.

	*An. aquasalis*	*An. darlingi*	*An. albimanus*	*An. gambiae*
CYP2	8	10	10	11
CYP3	32	32	35	39
CYP4	25	22	36	45
Mitochondrial CYP	8	8	8	9
Total	73	72	89	104

Evolutionary analyses (Additional file 1: Figure S9, S10 and S11) identified three *An. aquasalis* genes orthologous to *An. gambiae* genes related to insecticide resistance: 1921.g1856 is orthologous to AGAP002862 (CYP6AA1); 1805.g1684 related to AGAP008213 (CYP6M3)—both from the CYP3 family; and C559148.g12387, which is orthologous to AGAP001861-PA (CYP4H14)—from the CYP4 family. We also identified two losses of genes in the CYP2 family: the orthologous gene of CYP350 (AGAP005660, AALB015553, and ADAC003150) and the orthologous gene of CYP11179 (AGAP003065, AALB015657, ADAC007012). Finally, the *An. gambiae* mitochondrial CYP12F2 (AGAP008020-PA) and CYP12F3 (AGAP008019-PA) genes were not found in the genome of *An. aquasalis*.

Regarding glutathione-S-transferases, the genes were classified into seven classes: Delta, Epsilon, Omega, Sigma, Theta, Zeta, and Unclassified (Additional file 1: Table S5). The number of genes of the main classes Delta and Epsilon remained stable among the mosquitoes. Cholinesterases (CCEs) were classified into eight subfamilies: α-esterase, β-esterase, juvenile hormone esterase, acetylcholinesterase, gliotactin, glutactin, neurotactin and neuroligin (Additional file 1: Table S6). Among the identified CCEs, 36% belong to the α-esterase subfamily, the subfamily with the highest number of CCEs among anophelines (30–36%).

## Discussion

The sequencing and annotation of anopheline genomes allowed the identification of these mosquitoes’ structural, functional, and evolutionary differences. This is a task that started with *An. gambiae* at the beginning of this century and has so far included more than 15 species^29,31,32^. Five species of anophelines are responsible for most of the malaria transmission in the Americas. Two of them, *An. darlingi* and *An. albimanus* have already been sequenced. Here, we present the genome of the primary coastal malaria vector in Central and South America and the Caribbean Islands, *An. aquasalis.* Besides its importance as a vector of human malaria parasites, *An. aquasalis* has a remarkable ecological feature since its larvae grow in the brackish water of mangroves. Hence, its genomic sequences could reveal genes related to interaction with *Plasmodium* sp. and unique adaptations that allow it to deal with salted water, in contrast to other anophelines.

Anophelines have genomes ranging from 134.7 Mb (*An. darlingi*) to 375.8 Mb (*An. sinensis*)—with a median size of 224.3 Mb±50.3 Mb^29^. The number of genes ranges from 10,457 (*An. darlingi*) to 16,149 (*An. melas*), with a mean of 13,162 genes ± 1380^29^. Our results suggest that the size of the genome (162.9 Mb) and number of protein-coding genes (12,446) of *An. aquasalis* is relatively similar to other anophelines. We found a core of 1387 single-copy genes with orthologs in all Neotropical anophelines, *An. gambiae*, *An. merus*, *An. stephensi* and *D. melanogaster* and other Diptera. Based on these single-copy orthologs, we could reconstruct the evolutionary history of *An. aquasalis* and estimate its divergence time from other anophelines. Our data suggest that *An. aquasalis* diverged from *An. darlingi* approximately 20 mya^33,34^. The work of Martinez-Villegas^33^ was the first to estimate the divergence of *An. aquasalis* from other species using mitochondrial DNA data and found the divergence of *An. aquasalis* from *An. darlingi* to be ~ 39 mya. The results presented here are the first to calculate the divergence times of *An. aquasalis* to other anophelines based on a set of over a thousand nuclear genes and suggests not only that *An. aquasalis* is more closely related to *An. darlingi* than to *An. albimanus*, but also that both species have a much earlier divergence time than proposed by Martinez-Villegas.

The *An. aquasalis* repetitive elements and mobilome occupied of 6.28% of the genome, higher than that found in the American anophelines *An. albimanus* and *An. darlingi* ranges from 2.4 to 2.9% of the total genomic DNA. In general, repetitive elements in anophelines range from 0.13% in *An. koliensis* to 20% in *An. gambiae*^34,35^. However, the revision of repetitive elements in genomes assembled with long-read technologies has shown that the proportion of repetitive DNA is underestimated in many available genomes. Recent studies also found significant variations of repetitive elements in anopheline populations, such as that observed in *An. darlingi*, with repetitive elements varying from 2.9 to 5.6% of the genome^36,37^. Also, studies on the role of the mobilome in anophelines have shown the role of these DNA sequences in the modulation of immune response genes and detoxification genes^38^. All in all, the characterisation of repetitive elements and TE in anophelines has become a necessary process to understand how they are formed within the genome of these mosquitoes and as a possible explanation for the rapid response that some species have to unfavourable environmental factors and to the response to pathogens of medical interest^34,37,38^.

Insects have developed several mechanisms to live in saline environments, from regulation of ion transport and metabolism genes to morphological modifications of the rectum. We found 600 + proteins related to ion transport and metabolism, and our data suggest that *An. aquasalis* has a higher number of such proteins than other Neotropical anophelines and *An. gambiae*. Among the most abundant gene families related to ion transport, we identified many cation channels, zinc and copper transport and metabolism, and calcium binding, and transport proteins. *An. albimanus* and *An. merus* are other anophelines with tolerance to saline water, and both presented a lower number of such genes. Physiological studies have demonstrated that in the larvae of *An. albimanus*, when exposed to gradual changes in saline water, specialised non-dorsal anterior rectal (non-DAR) cells undergo changes in the localisation of V-type ATPases and K+/Na+ ATPases proteins, allowing the production of super osmotic urine and disrupting the ion reabsorption system in non-DAR cells^13,14^. Other studies of mosquitoes have also suggested the importance of carbonic anhydrase (CA) and aquaporins to osmoregulation in saline environments^15,17^. We found that *An. aquasalis* has a slightly higher number of such genes than *An. albimanus*. Interestingly, *An. merus* has a significantly higher number of canonical osmoregulation genes, especially in V-ATPases and K+/Na+ ATPases, with recent duplication events on these genes. It is possible that *An. aquasalis* and *An. merus* have different strategies to deal with saline environments, and further functional studies comparing both insects could reveal relevant adaptations for mosquito survivability in saline waters. We also hypothesised if the ability of *An. aquasalis* to live in saline water could be due to amino acid changes in such proteins (positive selection). However, as expected, our analysis suggests that all osmotic regulation genes are under strong purifying selection within and among orthologous groups in each protein family (the exception is the V-type ATPases in which each ortholog group seems to be evolving independently). Transcriptome studies in *An. merus* (another anopheline with high tolerance to saline environments)^16^ revealed several changes in gene expression upon salinity stress, raising a few candidates for further functional studies. Therefore, further transcriptome studies on *An. aquasalis* may reveal significant differences in gene expression and indicate candidates for functional studies.

*An. aquasalis* is one of the major malaria vectors in the New World, and studies in parasite-vector and vector-host interactions have been the focus of many researchers. For the *Plasmodium* to infect the anopheline, the parasite must overcome the insect’s immune system. The *An. aquasalis* genome revealed all genes from classical immune pathways in a one-to-one ortholog with *An. darlingi*. However, our study suggests that *An. aquasalis* (as the Neotropical *An. darlingi* and *An. albimanus*) has fewer immune-related genes than Old World anophelines.

In general, the family groups related to the control processes of reactive oxygen species (ROS) production and components regulating the expression of effectors of the immune response or signalling pathways were well conserved, with groupings of orthologs 1:1 for the four compared species. They are functionally relevant genes for the maintenance of the homeostasis of the organism and, in the case of signalling pathways such as the Toll pathway, they help in embryonic development processes and are constitutive for these insects^39–41^. On the other hand, the marked differences in the number of copies were in groups related to the recognition of molecular patterns of microorganisms, with sharp differences in the FREP and MLD proteins, especially with species-specific expansions in *An. gambiae*. A phenomenon that is possibly induced by the microbiota of each species or by the metabolic or sensory needs of each organism, as in the case of MLD proteins^42,43^.

Other families, such as PGRP or GNBP, had few differences in the number of copies, with losses mainly in American anophelines. These proteins activate signalling pathways, and some have been found as regulatory factors for other members of the same family^44,45^. The regulatory role of both families allowed a few variations to be maintained during evolution in American anophelines and *An. gambiae*. In addition, American mosquitoes suffered copy losses in cascade modulation proteins, mainly in serine proteases with CLIP domains, the most abundant family, and with gene expansions in *An. gambiae*^46^. These gene families activate signalling pathways and produce melanisation components through proteolytic cascades after recognising a pathogen^46,47^. It is recognised that specific sets of these proteins are organised for specific physiological and immune processes, sometimes with a redundant function that synergises to increase the intensity of the response^48,49^. In this sense, it is speculated how exposure to specific pathogens has shaped the set of serine proteases, serpins, and sometimes prophenoloxidase proteins in each species of mosquito^41,46^.

On the other spectrum, vector-host interactions, mosquitoes rely on a repertoire of chemosensory proteins to identify their hosts. Many studies have demonstrated that CSPs and OBPs present rapid evolution, sometimes limiting the identification of orthologs even in close species. The 16 *Anopheles* genomes manuscript suggested that most anophelines have ~ 60 OR copies, while all anophelines of the *gambiae* complex gained ~ 10 OR copies^29^. On the other hand, copy numbers of GRs and IRs have remained stable in all species studies so far. Our data suggest that *An. aquasalis* has a much lower number of OR and GR genes than other anophelines (44 and 32, respectively). As rapidly evolving genes, it is expected that the identification of OBPs is underestimated in genomes, which could be the reason for our findings. Neafsey and colleagues^29^ tried to find a correlation between OBP copy number variation (CNV) and host preference. However, transcriptome studies suggest that such differences are more likely due to functional divergence and regulation of gene expression^50,51^.

The increased resistance to insecticides in insect vectors of diseases is of significant concern for public health programs. Metabolic insecticide resistance is mediated by multi-copy gene families, such as cytochrome P450, glutathione S-transferases (GSTs), and carboxyl/cholinesterases. Despite its large numbers, several studies have shown the conservation of these gene families. We found 72 P450 in all relevant clades (CYP2, CYP3, CYP4, and mitochondrial CYP), similar to Neotropical anophelines. The same is true for GSTs and carboxyl/cholinesterases. In most cases, we found a 1:1 ortholog to *An. gambiae*, including orthologs with genes related to insecticide resistance, such as CYP6AA1, CYP4H14 (resistance to pyrethroids), CYP6M2 (resistance to carbamates), CYP6M3 (resistance to organochlorines)^52,53^ and GSTE2, GSTE4, and GSTE6 (resistance do DDT, organochlorines and pyrethroids, respectively)^54,55^. We also observed a few gene duplications and losses, and recent studies have suggested that CNV has a relevant role in the rise of pyrethroid resistance^56,57^. We have no reports on the increase in insecticide resistance in *An. aquasalis* and only a few studies have addressed this issue^58,59^. Identifying orthologs of genes related to major insecticide resistance is relevant for future studies on insecticide metabolism and evaluating insecticide resistance in natural populations.

## Conclusions

The data presented here brings new insights for *An. aquasalis* biology, Neotropical anopheline evolutionary relationships, and general anopheline evolution. Despite being the primary coastal malaria vector in Central and South America and the Caribbean Islands, the physiology of *An. aquasalis* still needs to be better understood. Recent research has elevated *An. aquasalis* as a significant model of vector-parasite interaction^19–21^, and the identification of immunity and digestion-related genes are essential for future research. Moreover, *An. aquasalis* is among the few anophelines capable of surviving drastic changes in water salinity, and with climate change and increased potential for saltwater invasion and salinisation of inland waters, studying the physiology of saltwater anophelines may be of great significance.

## Materials and methods

### *Anopheles aquasalis* mosquito sampling and sequencing

The mosquito sample used for this work came from the colony established in 1995 by the Laboratory of Medical Entomology at FIOCRUZ-MG. Genomic DNA was purified from a single adult female using a Qiagen DNeasy Kit for blood and tissues. The library was prepared using the Nextera DNA sample preparation kit (Epicentre Biotechnologies, Madison, WI), with an amplification step (5 cycles) as outlined in the Nextera protocol^61^. The fragment size distribution was analysed utilising a 2100 Bioanalyzer with a 7500 DNA assay kit (Agilent Technologies, Santa Clara, CA). Fragments of ~ 600 bp long were carried out for sequencing. The library was sequenced on one lane of an Illumina HiSeq2000 instrument to generate 50 base paired-end reads. Sequencing was performed by The Vincent J. Coates Genomics Sequencing Laboratory (GSL) at the University of California, Berkeley^60^. Sequences were assembled de novo using Velvet v1.2.10^61^ with a k-mer size of 41, according to the scripts and parameters suggested by the Velvet Manual (https://www.ebi.ac.uk/~zerbino/velvet/Manual.pdf) and in-house protocols from the UC Davis Vector Genetics Laboratory^61^. The assembled genome is available under the accession number GCA_002846955.1.

### Databases and sequences used to predict the genome of *An. aquasalis*

As part of the evidence implemented for gene prediction, cDNAs generated from the transcriptome of^24^ were downloaded from the GEO database with accession number GSE124997 on the NCBI website. In addition, proteins from four species of anophelines deposited in the VEuPathDB database were downloaded: *An. albimanus* (Anopheles-albimanus-STECLA_PEPTIDES_AalbS2.6), *An. darlingi* (Anopheles-darlingi-Coari_PEPTIDES_AdarC3.8.fa), *An. sinensis* (Anopheles-sinensis-China_PEPTIDES_AsinC2.2.fa) and *An. gambiae* (Anopheles-gambiae-PEST_PEPTIDES_AgamP4.12.fa)^62^.

### Annotation and prediction of repetitive elements in the genome of *An. aquasalis*

Simple repeats and complex repetitive elements in the genome of *An. aquasalis* were predicted using three approaches in the following order: 1st—we identified repeat elements using RepeatModeler (Version 2.0.4)^63^ with standard parameters; 2nd—we identified additional repeat elements using BLASTn against a database of repetitive elements from *An. gambiae*, *An darlingi*, *An. albimanus* and *An. stephensi* downloaded from VEuPathDB website^64^. 3rd—The repetitive elements identified with RepeatModeler and BLASTn were concatenated to build a database that was used to predict and mask repeat elements with RepeatMasker (Version 4.1.5)^64,65^. The options for searching the elements of interest were –nolow to mask only the interspaced sequences. –norna to not mask the smallRNA genes. The file with the program's predictions was obtained in gff format, with the –gff option^66^. The prediction results from all three steps were compared to build the final library of *An. aquasalis* repetitive elements.

### Structural prediction of the genome of *Anopheles aquasalis*

For structural prediction, a total of 60,752 proteins from five anopheline species are available in VEuPathDB and *An. aquasalis* transcripts^24^ were used as a template for gene annotation. Initial prediction of genes was performed with the MAKER program v. 2.31.1 available online on the Galaxy server^67^ in two rounds to create a draft of *An. aquasalis* putative genes. Using the option to infer gene predictions directly from all Expressed Sequenced Tags (from *An. aquasalis*), gene predictions were inferred directly from all protein alignments (database created), and pre-identified repeat elements from an external GFF file (repeat masker output). The gff output from MAKER, along with the unique scaffolds from the *An. aquasalis* genome was used to perform ab initio training with the annotator AUGUSTUS v. 3.3.3 in two rounds to create a draft of *An. aquasalis* putative genes. The MAKER archive and *An. aquasalis* ESTs were then used to train the AUGUSTUS program^68^. After training, a second round in MAKER was performed, using the same files as the first round with the addition of the gene models trained by AUGUSTUS. After this second round of MAKER, the gff output was used for AUGUSTUS second training, and then finally the final annotation by AUGUSTUS.and models generated by the training file were used for final annotation with AUGUSTUS using ESTs as evidence of transcription.

### Evaluation of *An. aquasalis* genome and annotation quality

The quality of the genome assembly and gene predictions were evaluated using the BUSCO v5.4.6, selecting the Diptera lineage and “genome assemblies” (for genome quality) or “protein” (for annotations) options on the Galaxy Australia server^67,69^. Additionally, the result obtained by the MAKER with the AED index was evaluated and established how much of the information used as evidence directly aligns with *An. aquasalis’* genome and this agreement between evidence and prediction must be equal to 90% of the generated alignments. The AED cumulative fraction of the annotations graph was generated using the AED_cdf_generator.pl script^70,71^.

### Functional prediction of the genome of *An. aquasalis*

Protein sequences originating from the *An. aquasalis* genome gene model was used for functional prediction, gene ontology (GO) assignments, and functional descriptions of the *An. aquasalis* genome was generated through the Pannzer program pipeline, selecting these annotations using ppv > 0.5^72^. Regarding protein domains and some gene ontologies (GO), these were annotated and searched for with the InterproScan tool from the Galaxy Europe server, selecting the annotations as an e-score of 0.0001^73^. The REVIGO tool (http://revigo.irb.hr/) was used to identify some GO terms not identified by functional annotation programs and to reduce the redundancy of these terms. Finally, a homology search was done using the Blastp program against the proteins of *Drosophila melanogaster* (Berkeley strain) (UP000000803), *Culex quinquefasciatus* (Southern House mosquito strain JHB) (UP000002320), *Aedes aegypti* (Yellow fever mosquito strain LVP_AGWV) (UP000008820), *An. darlingi* (UP000000673), *An. albimanus* (New World malaria mosquito strain STECLA/ALB19) (UP000069272) and *An. gambiae* (African malaria PEST strain) (UP000007062), choosing the sequences with a percentage identity > 50% and with an e-value of 0.0005^74^. The data generated by each tool were concatenated in Excel and the online Google Colaboratory tool and classified according to significance to determine the most accurate protein function. Basically, we established an order of priority for functional prediction with priority to Pannzer results followed by InterproScan, REVIGO, and Blastp (which means that we only assigned protein functionality based on Blastp in those cases that we had no results for Pannzer, InterproScan or REVIGO). To classify the terms of the genetic ontology obtained in the functional prediction, the R GO.db package (version 3.13.0) was used, using the option “GOANCESTOR”. The functional prediction was complemented with orthology searches in OrthoDB (v11) at the Diptera level^75^.

### Orthology analysis

Orthology assignments were retrieved from OrthoDB (v11)^75^ Diptera-level orthologous groups (116 species) for the species detailed in Dataset S1. *An. aquasalis* protein-coding genes were mapped to OrthoDB (v11) at the Diptera level using *D. melanogaster* as an anchor. Mapping was also performed for *An. gambiae, An. darlingi, An. albimanus, An. stephensi*, *An. merus*, *A. aegypti* and *D. melanogaster*. Each species was then merged to create the final orthologous groups, including all mapped *An. aquasalis* proteins. Single copy genes were then identified for all species to build a dataset of genes for phylogenomics. The presence, absence, and copy-numbers of orthologs were also assessed to partition genes from each Dipteran species into the categories shown in the bar chart (Fig. 1A) and classified as: (1) single copy orthologs present in all taxa; (2) multicopy orthologs present in all taxa; (3) orthologs present in *D. melanogaster* and at least one mosquito; (4) orthologs present in all mosquitoes; (5) orthologs present in other Diptera; (6) single-copy orthologs present in all anophelines; (7) orthologs present in more than one anopheline (but not all); (8) orthologs present only in Neotropical anophelines; (9) Orthologs present only in old-world anophelins; (11) proteins with no orthology at Diptera level. A Venn diagram was created with the VennDiagram package for R^76^.

### Phylogenomics and gene evolutionary analysis

All evolutionary analyses were conducted in MEGA 11^77^. For the main evolutionary analysis of *An. aquasalis*, a total of 1387 single-copy orthologous proteins from *An. aquasalis, An. darlingi, An. albimanus, An. gambiae, An. merus, An. stephensi, Ae. aegypti* and *D. melanogaster* (outgroup) were used. Proteins were aligned with MUSCLE and the phylogenetic tree constructed by Maximum Likelihood (with 10,000 bootstrap replications, JTT model, Uniform Rates and Complete Deletion). Divergence times were inferred by the RelTime method^78,79^ specifying *T. castaneum* as the outgroup and using three fixed calibration constraints based on data available at http://www.timetree.org/^80^, being: *D. melanogaster* to *An. gambiae* (241 mya); *Ae. aegypti* to *An. gambiae* (149 mya) and; *An. darlingi* to *An. gambiae* (79 mya).

Protein-coding sequences were translated to amino acids for the evolutionary analysis and substitution rates of osmoregulation proteins and aligned using Muscle v5^81^. Phylogenetic trees were constructed using amino acid sequences, using neighbour-joining (for orthology analysis)^82^ with 10,000 replicates and a complete deletion option^77,78^. Purifying and positive selection hypotheses were tested via synonymous and nonsynonymous substitutions per site (dS and dN, respectively) in MEGA11. P values less than 0.05 were considered significant at the 5% level within and among orthologous groups for each gene family. The variance of the difference was computed using the analytical method. Analyses were conducted using the Nei-Gojobori method^83^. For other simpler evolutionary analyses, phylogenetic trees were constructed using amino acid sequences aligned by Muscle, using neighbour-joining (for orthology analysis)^82^ with 10,000 replicates and a complete deletion option.

### Heatmap analysis of the number of ion transport/metabolism and osmoregulation genes

The number of orthologous genes was retrieved from Additional File 3—Dataset 2 and clustered in family functions. The Heatmap was constructed using the Heatmapper software^84^ clustered by average linkage and Euclidean distance measurement method, applying clustering to rows and columns.

### Supplementary Information

Supplementary Information 1.

Supplementary Information 2.

Supplementary Information 3.

Supplementary Information 4.

Supplementary Information 5.

Supplementary Information 6.

## Data Availability

The *Anopheles aquasalis* genome is deposited in GeneBank under accession GCA_002846955.1 (https://www.ncbi.nlm.nih.gov/data-hub/genome/GCA_002846955.1/). Annotated proteins are under submission to GeneBank and can be downloaded as Additional File 4 (CDS) and Additional File 5 (Proteins).
