# Supplementary material for: Unravelling the genome of the brackish water malaria vector Anopheles aquasalis
Source: Sci Rep. 2023 Nov 22;13:20472. doi: 10.1038/s41598-023-47830-1 (PMC10665375; doi:10.1038/s41598-023-47830-1)
Supplement: Supplementary file 1 — Supplementary Information 1. [file 41598_2023_47830_MOESM1_ESM.docx]

Supplementary Information for:

**Unravelling the genome of the brackish water malaria vector *Anopheles aquasalis***

Cesar C. Prado-Sepulveda^1,2&^, Rodrigo M. Alencar^1,2&^, Luis Martinez-Villegas^3^, Ana Cristina Bahia^6^, Rosa A. Santana^1,2^, Igor B. de Souza^1,2^, Gigliola M. A. D’Elia^1,2^, Ana Paula M. Duarte^1,2^, Marcus V. G. de Lacerda^1,4^, Wuelton M. Monteiro^1^, Nágila F. Costa Secundino^1,2,3^, Leonardo B. Koerich^5^*, Paulo F. P. Pimenta^1,2,3^*

^1^ Fundação de Medicina Tropical Dr. Heitor Vieira Dourado, Manaus, Amazonas, Brasil. CEP 69.040-000

^2^ Programa de Pós-Graduação em Medicina Tropical, Fundação de Medicina Tropical Heitor Vieira Dourado, Universidade do Estado do Amazonas, Manaus, Amazonas, Brasil. CEP69.040-000

^3^ Instituto de Pesquisas René Rachou, Fundação Oswaldo Cruz, Belo Horizonte, Minas Gerais, Brasil. CEP 30.190-009

^4^ Instituto de Pesquisas Leônidas e Maria Deane, Fundação Oswaldo Cruz, Manaus, Amazonas, Brasil. CEP 69.027-070

^5^ Departamento de Parasitologia, Universidade Federal de Minas Gerais, Belo Horizonte, Minas Gerais, Brasil. CEP 31.270-901

^6^Laboratório de Bioquímica de Insetos e Parasitos, Instituto de Biofísica Carlos Chagas Filho, Universidade Federal do Rio de Janeiro, Rio de janeiro, CEP 21.941-170

^&^C.C.P.S. and R.M.A. contributed equally to this work

*Corresponding authors: Leonardo B. Koerich and Paulo F. P. Pimenta

**Email:**  [lbkoerich@ufmg.br;](mailto:lbkoerich@ufmg.br;) [pfppimenta@gmail.com](mailto:pfppimenta@gmail.com)


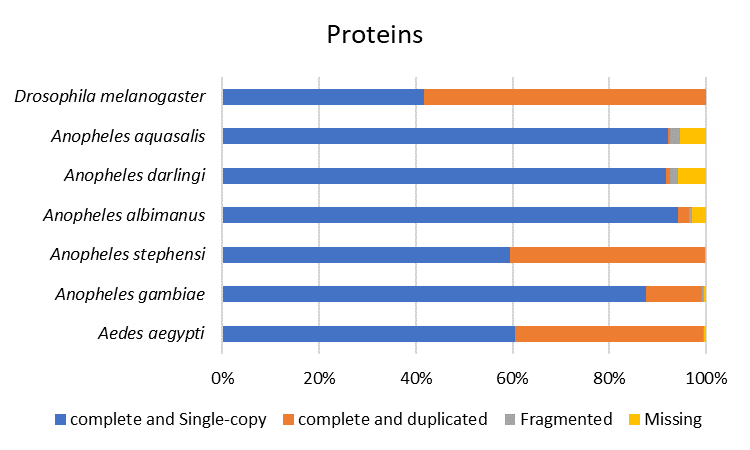


**
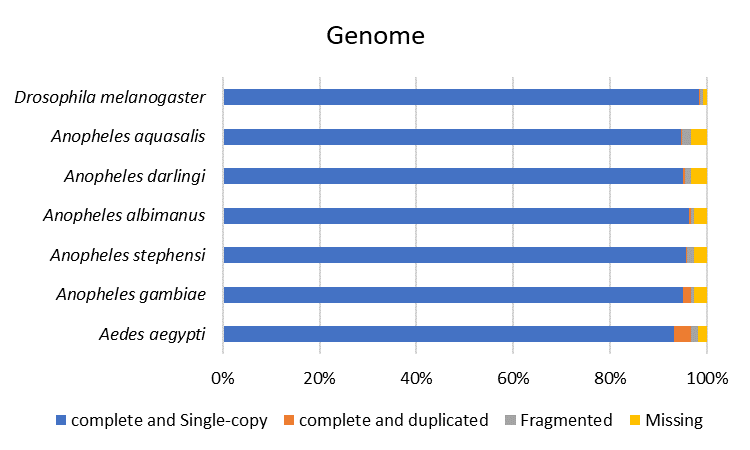
**

**Figure S1.** BUSCOs comparison of the integrity of the genomes and proteomes of *Anopheles aquasalis* plus six dipteran species.


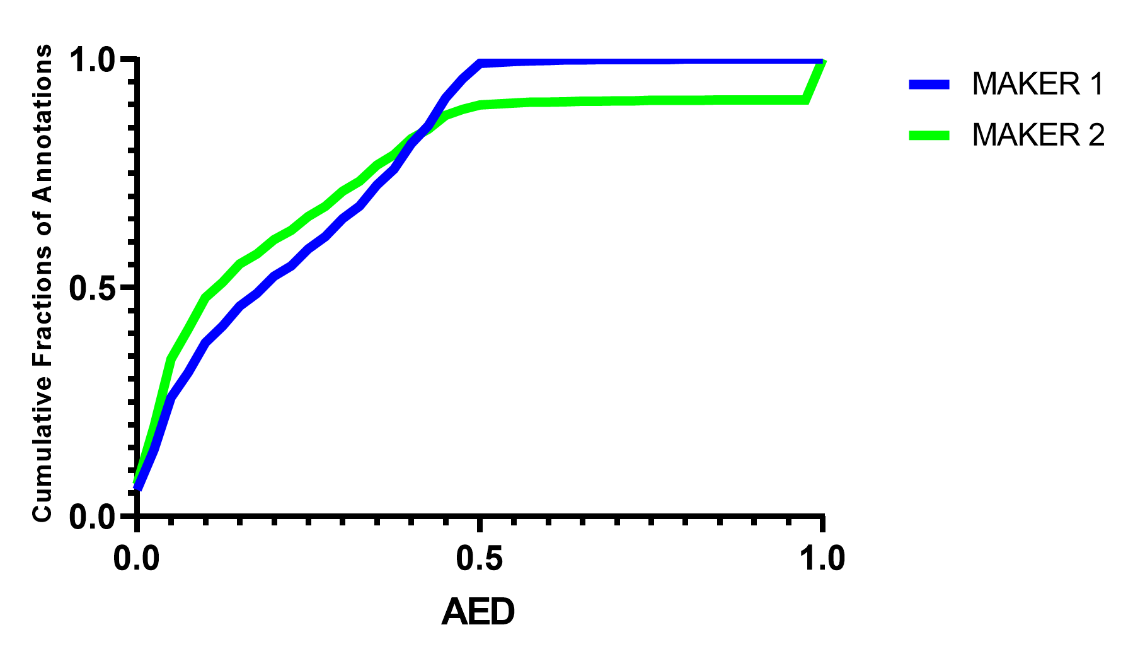


**Figure S2.** Evaluation of the annotation of the gene model generated by the MAKER program with the annotation edit distance index (AED). In blue is the result of the first annotation and in green is the result of the same index for the second annotation.


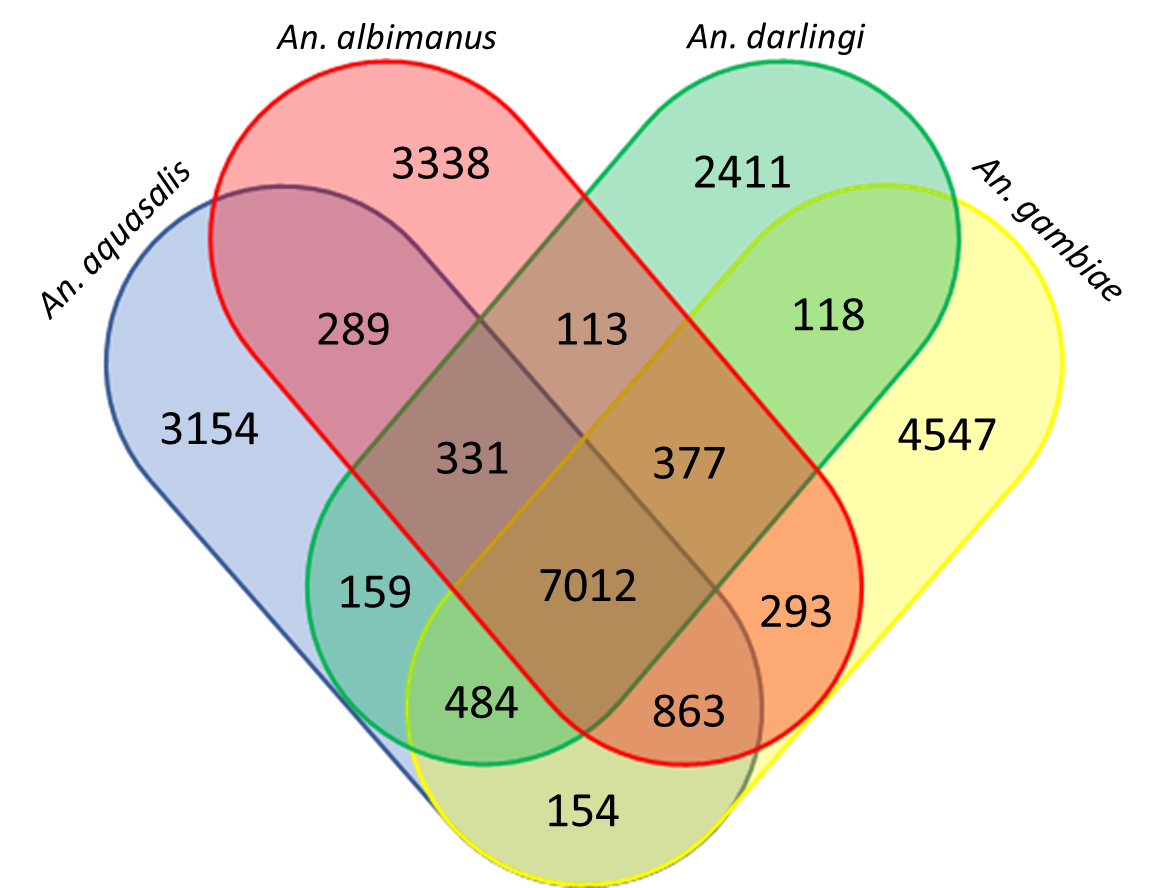


**Figure S3. Venn diagram of orthologs.** The Venn diagram shows the clustering of *An. aquasalis* genes (blue), identified by OrthoDB, in relation to three other anopheline species: *An. darlingi* (green); *An. albimanus* (red) and *An. gambiae* (yellow).


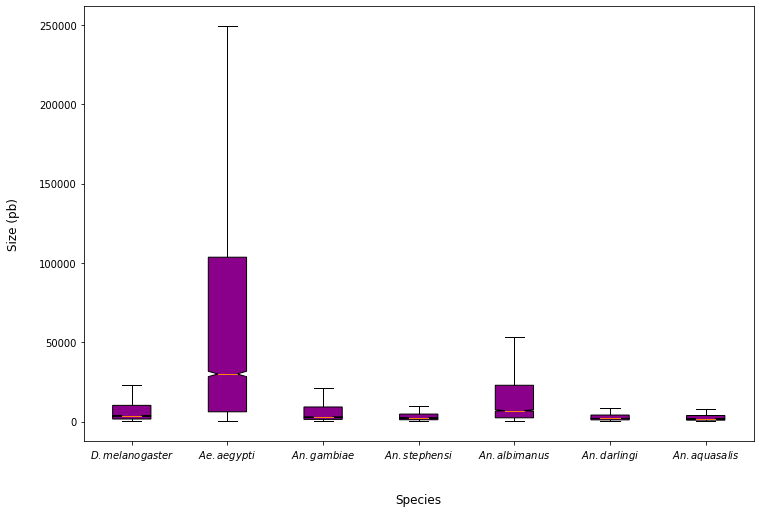

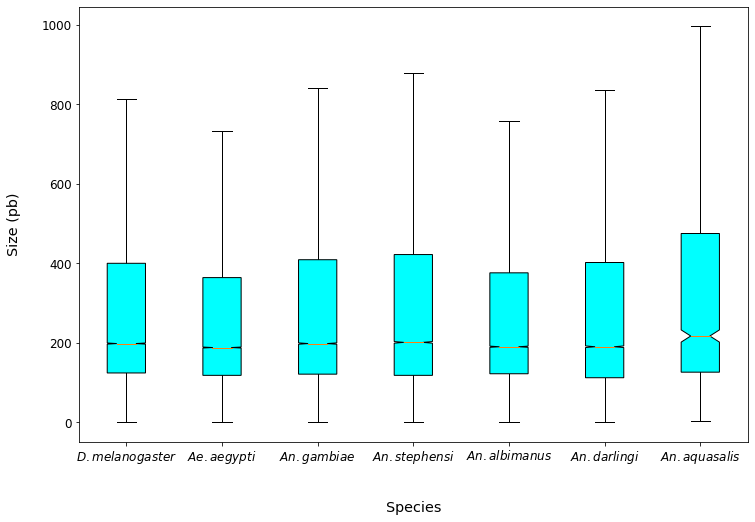


**B**

**A**

**Figure S4.** Structural comparison of genes among seven species of Diptera. A) Comparison of transcript size among seven species of Diptera. B) Comparison of the size of the coding region (CDS) among seven species of Diptera.


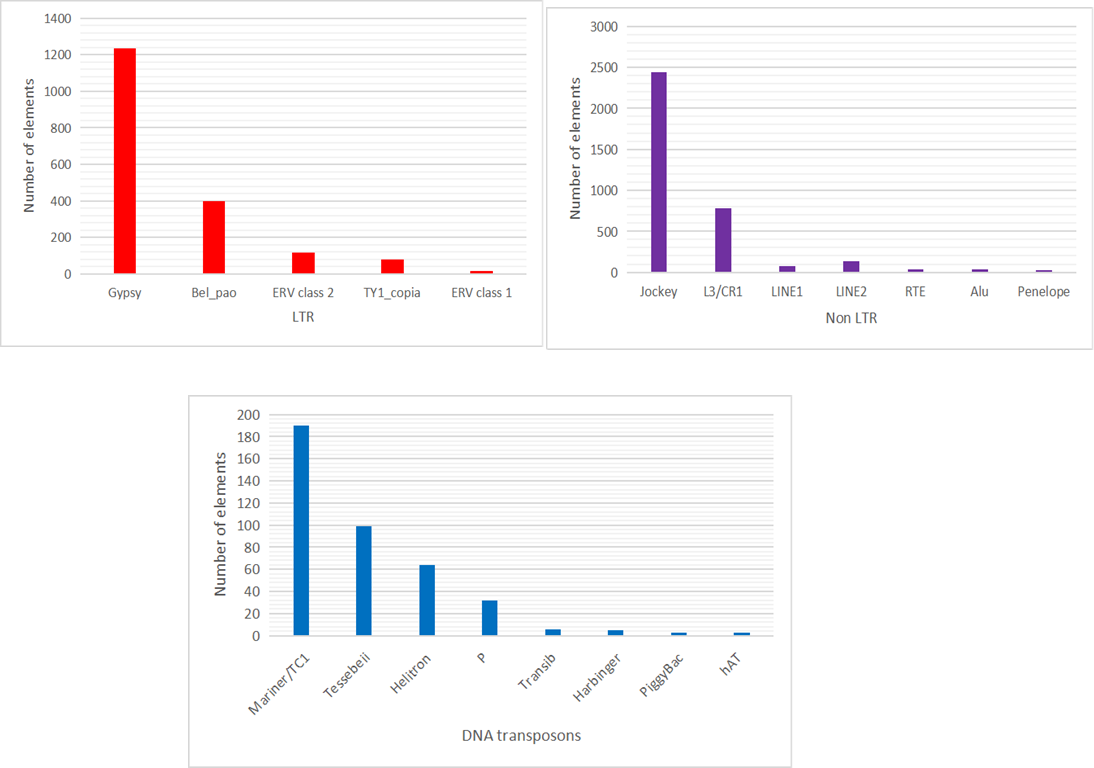


**Figure S5.** Transposable elements families in *An. aquasalis*.

**Table S1.** General structural features of *An. aquasalis* genes

|  | **Max.** | **Min.** | **Mean** | **Median** |
| --- | --- | --- | --- | --- |
| CDS | 13,218 | 3 | 420.88 | 211 |
| Transcript | 63,633 | 201 | 4059.96 | 1983 |
| Intron | 32,585 | 55 | 684.4 | 84 |
| Gene | 63,632 | 200 | 3508.37 | 1742.5 |

The composition of the repetitive elements in the genome of *An. aquasalis* is 0.95%. Of these elements found in the genome of *An. aquasalis*, 0.40% were identified as retroelements and 0.21% as DNA transposons. The remaining 0.34% of the sequences were not categorized, so they were removed from the masking. Among the transposable elements in *An. aquasalis*, most corresponded to the LINEs (30.55%), followed by the DNA transposable elements (22.3%) and, finally, the long terminal LTR repeats (11.22%) of the total finding with the tool using homology (Table S1).

**Table S2.** Composition of the DNA repetitive sequences of *An. aquasalis*.

| **Elements** | **Number of elements** | **Length** | **Percentage** |
| --- | --- | --- | --- |
| **Retroelements** | 7,290 | 503,867 bp | **0.40%** |
| **SINEs:** | 4 | 220 bp | 0.00% |
| **Penelope** | 1 | 22 bp | 0.00% |
| **LINEs:** | 5,726 | 368,137 bp | 0.29% |
| **CRE/SLACS** | 0 | 0 bp | 0.00% |
| **L2/CR1/Rex** | 80 | 7,976 bp | 0.01% |
| **R1/LOA/Jockey** | 388 | 30,028 bp | 0.02% |
| **R2/R4/NeSL** | 7 | 417 bp | 0.00% |
| **RTE/Bov-B** | 44 | 7,510 bp | 0.01% |
| **L1/CIN4** | 119 | 5,426 bp | 0.00% |
| **LTR elements:** | 1,560 | 135,510 bp | 0.11% |
| **BEL/Pao** | 173 | 32,413 bp | 0.03% |
| **Ty1/Copia** | 43 | 2,895 bp | 0.00% |
| **Gypsy/DIRS1** | 1,341 | 99,877 bp | 0.08% |
| **Retroviral** | 0 | 0 bp | 0.00% |
| **DNA transposons** | 3,837 | 269,843 bp | **0.21%** |
| **hobo-Activator** | 2 | 183 bp | 0.00% |
| **Tc1-IS630-Pogo** | 12 | 800 bp | 0.00% |
| **En-Spm** | 0 | 0 bp | 0.00% |
| **MuDR-IS905** | 0 | 0 bp | 0.00% |
| **PiggyBac** | 1 | 40 bp | 0.00% |
| **Tourist/Harbinger** | 5 | 533 bp | 0.00% |
| **Other (Mirage,P-element, Transib)** | 54 | 4,486 bp | 0.00% |
| **Rolling-circles** | 29 | 4,116 bp | 0.00% |
| **Unclassified** | 4,151 | 433,291 bp | 0.34% |
| **Total interspersed repeats** |  | **1,207,001 bp** | **0.95%** |

**Table S3.** Most abundant domains in the New World anopheline species *An. aquasalis*, *An. darlingi* and *An. albimanus.*

|  |  | Number of proteins | | |
| --- | --- | --- | --- | --- |
| InterPro ID | Description | *An. aquasalis* | *An. darlingi* | *An. albimanus* |
| IPR013087 | Zinc finger C2H2-type | 301 | 246 | 285 |
| IPR000719 | Protein kinase domain | 212 | 207 | 188 |
| IPR001254 | Serine proteases, trypsin domain | 200 | 195 | 188 |
| IPR013083 | Zinc finger, RING/FYVE/PHD-type | 196 | 182 | 191 |
| IPR007110 | Immunoglobulin-like domain | 168 | 142 | 175 |
| IPR001611 | Leucine-rich repeat | 163 | 144 | 150 |
| IPR001680 | WD40 repeat | 160 | 159 | 170 |
| IPR001314 | Peptidase S1A, chymotrypsin family | 154 | 157 | 144 |
| IPR003591 | Leucine-rich repeat, typical subtype | 122 | 103 | 112 |
| IPR000504 | RNA recognition motif domain | 120 | 115 | 119 |
| IPR003593 | AAA+ ATPase domain | 116 | 111 | 115 |
| IPR001841 | Zinc finger, RING-type | 101 | 96 | 99 |
| IPR000618 | Insect cuticle protein | 97 | 91 | 93 |
| IPR002557 | Chitin-binding domain | 96 | 52 | 87 |
| IPR002110 | Ankyrin repeat | 92 | 85 | 99 |
| IPR001356 | Homeobox domain | 90 | 58 | 82 |
| IPR001128 | Cytochrome P450 | 87 | 93 | 99 |
| IPR000276 | G protein-coupled receptor, rhodopsin-like | 87 | 68 | 99 |
| IPR012934 | Zinc finger, AD-type | 78 | 76 | 89 |
| IPR001650 | Helicase, C-terminal | 77 | 75 | 80 |
| IPR000210 | BTB/POZ domain | 73 | 60 | 65 |
| IPR002048 | EF-hand domain | 73 | 70 | 77 |
| IPR001478 | PDZ domain | 72 | 61 | 70 |
| IPR001849 | Pleckstrin homology domain | 66 | 68 | 73 |
| IPR003961 | Fibronectin type III | 64 | 63 | 67 |
| IPR001806 | Small GTPase | 60 | 57 | 58 |
| IPR001452 | SH3 domain | 60 | 64 | 65 |
| IPR006170 | Pheromone/general odorant-binding protein | 55 | 32 | 40 |
| IPR003439 | ABC transporter-like, ATP-binding domain | 54 | 55 | 48 |
| IPR011701 | Major facilitator superfamily | 53 | 56 | 56 |
| IPR001245 | Serine-threonine/tyrosine-protein kinase, catalytic domain | 49 | 54 | 35 |
| IPR001251 | CRAL-TRIO lipid-binding domain | 48 | 49 | 37 |


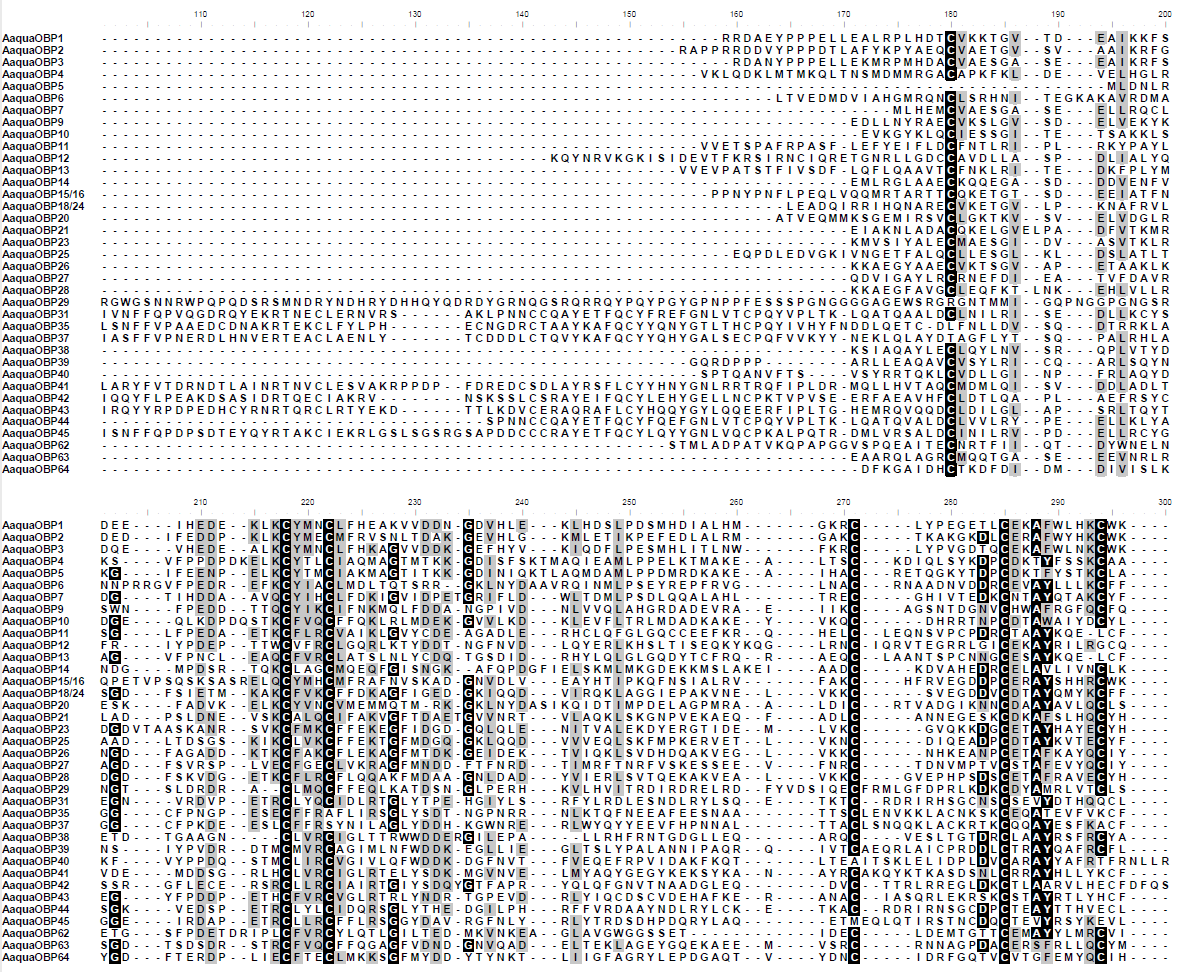


**Figure S6.** Multiple alignments of *An. aquasalis* OBPs to identify conserved cysteine residues.

**Table S4.** OBP genes found in the genome of *An. aquasalis*

| **Query** | **Aaqua OBP** | **Subfamily** | **Size CDS** | **Size aa** | **Signal P start** | **Signal P end** | **CDD short name** | **CDD Superfamily** | **PFAM INTERPRO** |
| --- | --- | --- | --- | --- | --- | --- | --- | --- | --- |
| 2978.g3569 | AaquaOBP43 | Atypical | 1023 | 340 | 1 | 23 | PBP_GOBP | cl11600 | PBP/GOBP family |
| 8779.g11276 | AaquaOBP9 | Classic | 420 | 139 | 1 | 17 | PBP_GOBP | cl11600 | PBP/GOBP family |
| 8493.g10819 | AaquaOBP63 | Classic | 357 | 118 | #N/A | #N/A | PBP_GOBP | cl11600 | PBP/GOBP family |
| 4149.g5178 | AaquaOBP31 | Atypical | 1275 | 424 | 1 | 22 | PBP_GOBP | cl11600 | PBP/GOBP family |
| 4149.g5179 | AaquaOBP45 | Atypical | 1362 | 453 | 1 | 26 | PBP_GOBP | cl11600 | PBP/GOBP family |
| 8493.g10820 | AaquaOBP26 | Classic | 396 | 131 | 1 | 18 | PBP_GOBP | cl11600 | PBP/GOBP family |
| 6912.g8608 | AaquaOBP41 | Atypical | 858 | 285 | 1 | 29 | PBP_GOBP | cl11600 | PBP/GOBP family |
| 1215.g336 | AaquaOBP39 | Atypical | 891 | 296 | 1 | 32 | PBP_GOBP | cl11600 | PBP/GOBP family |
| C493700.g12054 | AaquaOBP35 | Atypical | 831 | 276 | 1 | 20 | PBP_GOBP | cl11600 | PBP/GOBP family |
| 8493.g10822 | AaquaOBP18/24 | Classic | 471 | 156 | #N/A | #N/A | PBP_GOBP | cl11600 | PBP/GOBP family |
| 8493.g10816 | AaquaOBP28 | Classic | 417 | 138 | 1 | 20 | PBP_GOBP | cl11600 | PBP/GOBP family |
| 6459.g8135 | AaquaOBP15/16 | Classic | 516 | 171 | #N/A | #N/A | PBP_GOBP | cl11600 | PBP/GOBP family |
| 2191.g2289 | AaquaOBP42 | Atypical | 966 | 321 | 1 | 21 | PBP_GOBP | cl11600 | PBP/GOBP family |
| 8493.g10821 | AaquaOBP25 | Classic | 447 | 148 | 1 | 20 | PBP_GOBP | cl11600 | PBP/GOBP family |
| 1386.g768 | AaquaOBP20 | Classic | 483 | 160 | 1 | 39 | PBP_GOBP | cl11600 | #N/A |
| 8493.g10823 | AaquaOBP23 | Classic | 414 | 137 | 1 | 22 | PBP_GOBP | cl11600 | PBP/GOBP family |
| 3948.g4896 | AaquaOBP1 | Classic | 555 | 184 | 1 | 25 | PBP_GOBP | cl11600 | PBP/GOBP family |
| 8851.g11407 | AaquaOBP3 | Classic | 468 | 155 | 1 | 31 | PBP_GOBP | cl11600 | PBP/GOBP family |
| 1798.g1678 | AaquaOBP10 | Classic | 414 | 137 | 1 | 22 | PBP_GOBP | cl11600 | PBP/GOBP family |
| 2824.g3290 | AaquaOBP37 | Atypical | 897 | 298 | 1 | 22 | PBP_GOBP | cl11600 | PBP/GOBP family |
| 6459.g8134 | AaquaOBP2 | Classic | 498 | 165 | #N/A | #N/A | PBP_GOBP | cl11600 | PBP/GOBP family |
| 460.g5824 | AaquaOBP21 | Classic | 420 | 139 | 1 | 21 | PBP_GOBP | cl11600 | PBP/GOBP family |
| 1215.g334 | AaquaOBP12 | Classic | 741 | 246 | 1 | 22 | PBP_GOBP | cl11600 | PBP/GOBP family |
| 1863.g1758 | AaquaOBP4 | Classic | 543 | 180 | #N/A | #N/A | PBP_GOBP | cl11600 | PBP/GOBP family |
| 5301.g6641 | AaquaOBP6 | Classic | 486 | 161 | 1 | 27 | PBP_GOBP | cl11600 | PBP/GOBP family |
| 8150.g10277 | AaquaOBP7 | Classic | 339 | 112 | #N/A | #N/A | PBP_GOBP | cl11600 | PBP/GOBP family |
| 8973.g11644 | AaquaOBP14 | PlusC* | 432 | 143 | 1 | 20 | PBP_GOBP | cl11600 | PBP/GOBP family |
| 1215.g337 | AaquaOBP40 | Atypical | 1041 | 346 | 1 | 36 | PBP_GOBP | cl11600 | PBP/GOBP family |
| 397.g4962 | AaquaOBP5 | Classic | 291 | 96 | #N/A | #N/A | PBP_GOBP | cl11600 | PBP/GOBP family |
| 8493.g10817 | AaquaOBP64 | Classic | 426 | 141 | 1 | 28 | PBP_GOBP | cl11600 | PBP/GOBP family |
| 8493.g10818 | AaquaOBP27 | Classic | 414 | 137 | 1 | 25 | PBP_GOBP | cl11600 | PBP/GOBP family |
| 651.g8208 | AaquaOBP38 | Atypical | 1227 | 408 | 1 | 25 | PBP_GOBP | cl11600 | PBP/GOBP family |
| 1733.g1521 | AaquaOBP11 | Classic | 588 | 195 | 1 | 23 | PBP_GOBP | cl11600 | PBP/GOBP family |
| 122.g355 | AaquaOBP20_2 | PlusC* | 2256 | 751 | 1 | 39 | PBP_GOBP | cl11600 | #N/A |
| 1215.g335 | AaquaOBP13 | Classic | 573 | 190 | 1 | 21 | PBP_GOBP | cl11600 | PBP/GOBP family |
| 4149.g5177 | AaquaOBP44 | Atypical | 873 | 290 | #N/A | #N/A | PBP_GOBP | - | PBP/GOBP family |
| 157.g1071 | AaquaOBP157.g1071 | Classic | 837 | 278 | #N/A | #N/A | #N/A | #N/A | #N/A |
| 4433.g5610 | AaquaOBP62 | Classic | 501 | 166 | 1 | 26 | PBP_GOBP | - | PBP/GOBP family |
| 1180.g281 | AaquaOBP29 | Classic | 900 | 299 | 1 | 24 | PBP_GOBP | cl11600 | PBP/GOBP family |
| 5143.g6430 | AaquaOBP5143.g6430 | Classic | 948 | 315 | #N/A | #N/A | #N/A | #N/A | #N/A |
| 261.g3012 | AaquaOBP57 | PlusC | 504 | 167 | #N/A | #N/A | #N/A | #N/A | PBP/GOBP family |
| 6154.g7740 | AaquaOBP80 | PlusC | 618 | 205 | 1 | 29 | #N/A | #N/A | PBP/GOBP family |
| 3241.g3964 | AaquaOBP46 | PlusC | 624 | 207 | 1 | 24 | #N/A | #N/A | PBP/GOBP family |
| 3241.g3961 | AaquaOBP61 | PlusC | 603 | 200 | 1 | 19 | #N/A | #N/A | #N/A |
| 3241.g3960 | AaquaOBP60 | PlusC | 597 | 198 | 1 | 18 | #N/A | #N/A | #N/A |
| 3241.g3966 | AaquaOBP47 | PlusC | 582 | 193 | 1 | 21 | #N/A | #N/A | #N/A |
| 5805.g7304 | AaquaOBP58 | PlusC | 795 | 264 | 1 | 21 | #N/A | #N/A | #N/A |
| 3241.g3967 | AaquaOBP48 | PlusC | 591 | 196 | 1 | 25 | #N/A | #N/A | #N/A |
| 157.g1070 | AaquaOBP157.g1070 | Classic | 705 | 234 | #N/A | #N/A | #N/A | #N/A | #N/A |
| 3241.g3962 | AaquaOBP66 | Classic* | 525 | 174 | #N/A | #N/A | #N/A | #N/A | PBP/GOBP family |


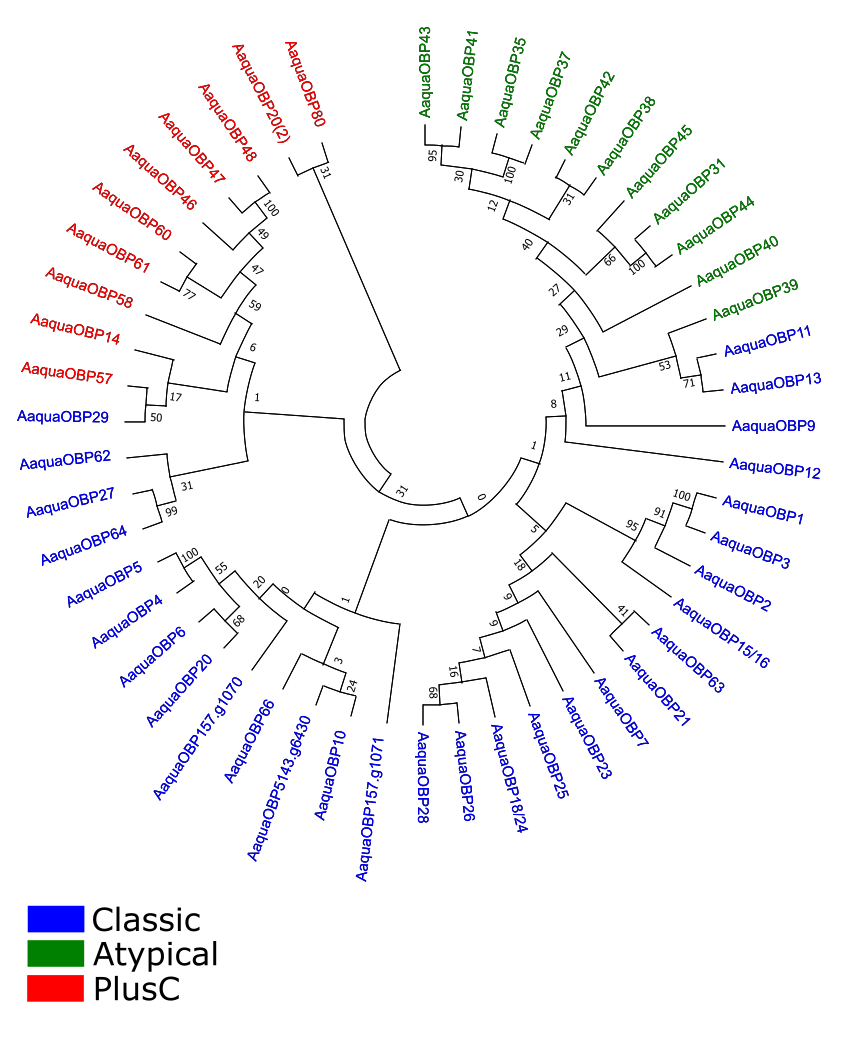


**Figure S7.** Neighbor-joining tree of OBP amino acid sequences of *An. aquasalis.* Classification into Classic, Atypical and PlusC subfamilies


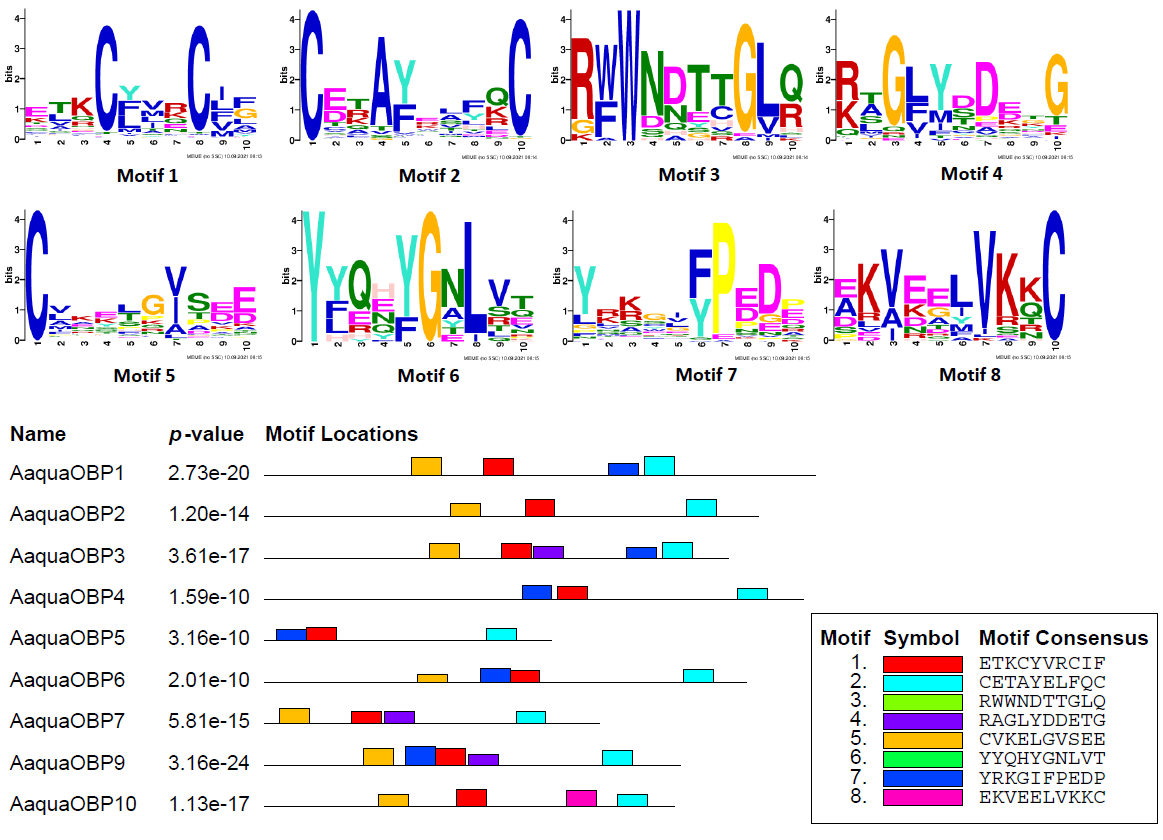


**Figure S8.** Analysis of OBP motifs from *An. aquasalis, An. gambiae, An. darlingi* and *An. albimanus.*


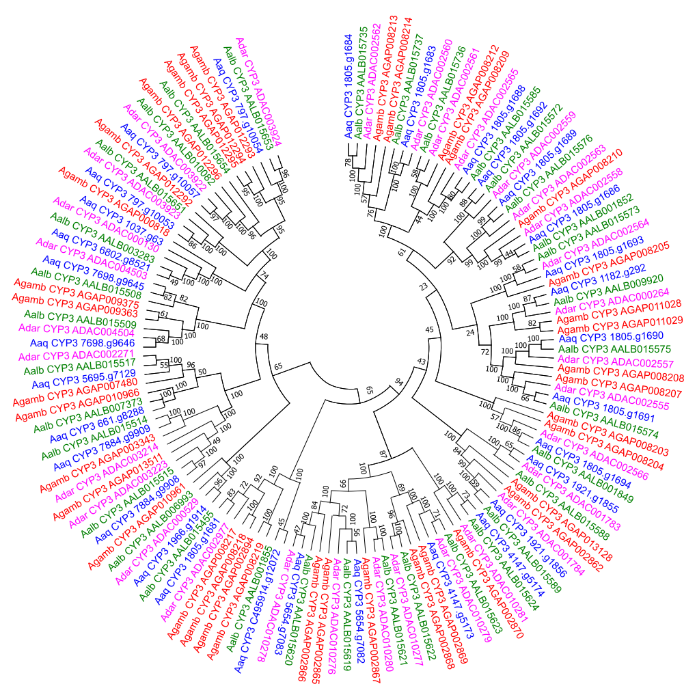


**Figure S9.** Neighbor-joining tree of CYP3 amino acid sequences of *An. aquasalis* (blue), *An. darlingi* (pink), *An. albimanus* (green) and *An. gambiae* (red). Bootstrap values were calculated with 1,000 replicates, and their values were presented in each branch of the tree.


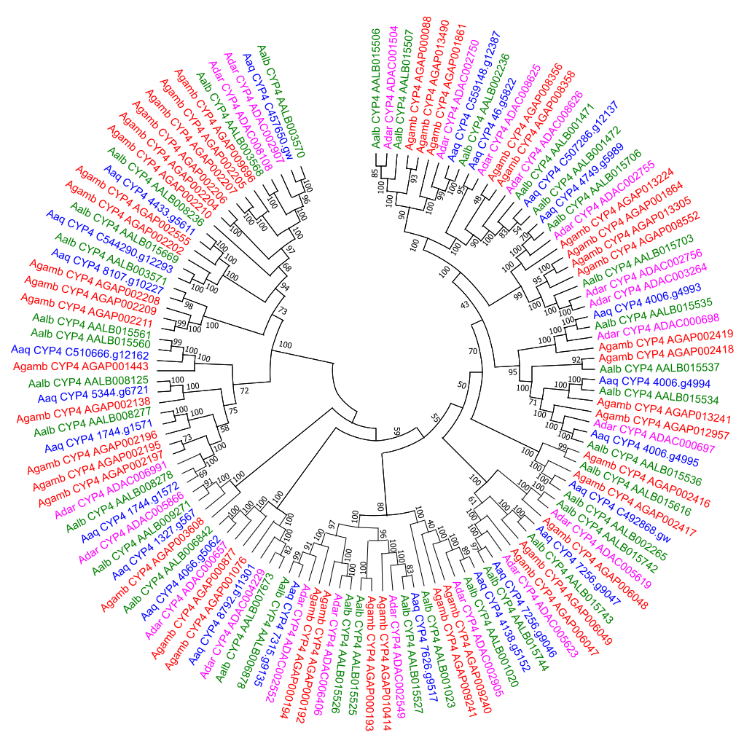


**Figure S10.** Neighbor-joining tree of CYP4 amino acid sequences of *An. aquasalis* (blue), *An. darlingi* (pink), *An. albimanus* (green) and *An. gambiae* (red). Bootstrap values were calculated with 1,000 replicates and their values were presented in each branch of the tree.


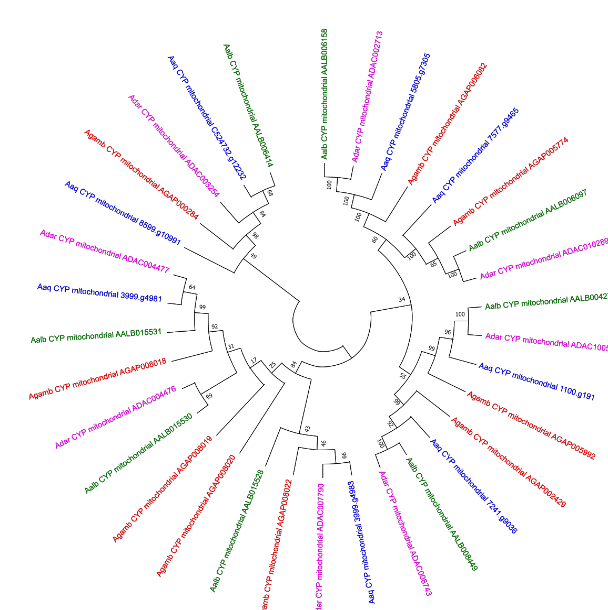

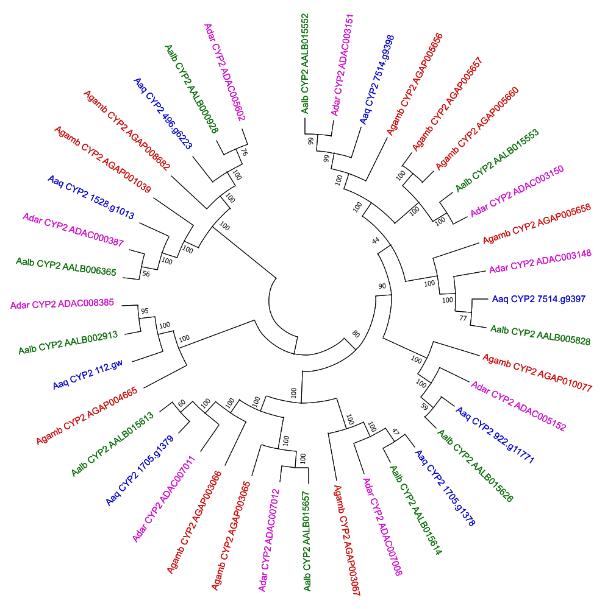

**Figure S11.** Neighbor-joining tree of CYP2 (A) and mitochondrial CYP (B) amino acid sequences of *An. aquasalis* (blue), *An. darlingi* (pink), *An. albimanus* (green) and *An. gambiae* (red). Bootstrap values were calculated with 1,000 replicates and their values were presented in each branch of the tree.

**Table S5.** *An. aquasalis* glutathione-S-transferases gene subfamilies and comparison with *An. darlingi*, *An. albimanus* and *An. gambiae.*

|  | *An. aquasalis* | *An. darlingi* | *An. albimanus* | *An. gambiae* |
| --- | --- | --- | --- | --- |
| Delta | 11 | 11 | 10 | 11 |
| Epsilon | 5 | 6 | 6 | 6 |
| Omega | 1 | 1 | 1 | 1 |
| Sigma | 2 | 2 | 1 | 2 |
| Theta | 2 | 2 | 2 | 2 |
| Zeta | 1 | 1 | 1 | 1 |
| Unclassified | 3 | 3 | 3 | 3 |
| **Total** | **25** | **26** | **24** | **26** |

**Table S6.** *An. aquasalis* cholinesterases gene subfamilies and comparison with *An. darlingi*, *An. albimanus* and *An. gambiae.*

|  | *An. aquasalis* | *An. darlingi* | *An. albimanus* | *An. gambiae* |
| --- | --- | --- | --- | --- |
| α-Esterase | 13 | 14 | 11 | 14 |
| β-Esterase | 2 | 3 | 1 | 5 |
| Juvenile hormone | 3 | 4 | 2 | 5 |
| Acetylcholinesterase | 2 | 2 | 2 | 2 |
| Uncharacterized | 1 | 1 | 1 | 1 |
| Gliotactin | 1 | 1 | 1 | 1 |
| Glutactin | 5 | 5 | 3 | 7 |
| Uncharacterized | 2 | 1 | 2 | 3 |
| Neurotactin | 2 | 2 | 2 | 2 |
| Uncharacterized H | 1 | 1 | 1 | 1 |
| Neuroglin | 4 | 5 | 4 | 4 |
| **Total** | **36** | **39** | **30** | **45** |
